# Diffusiophoretic Behavior of Polyelectrolyte-Coated Particles

**DOI:** 10.1021/acs.langmuir.3c03916

**Published:** 2024-03-07

**Authors:** Burak Akdeniz, Jeffery A. Wood, Rob G. H. Lammertink

**Affiliations:** Soft Matter, Fluidics and Interfaces, MESA+ Institute for Nanotechnology, University of Twente, P.O. Box 217, Enschede 7500 AE, The Netherlands

## Abstract

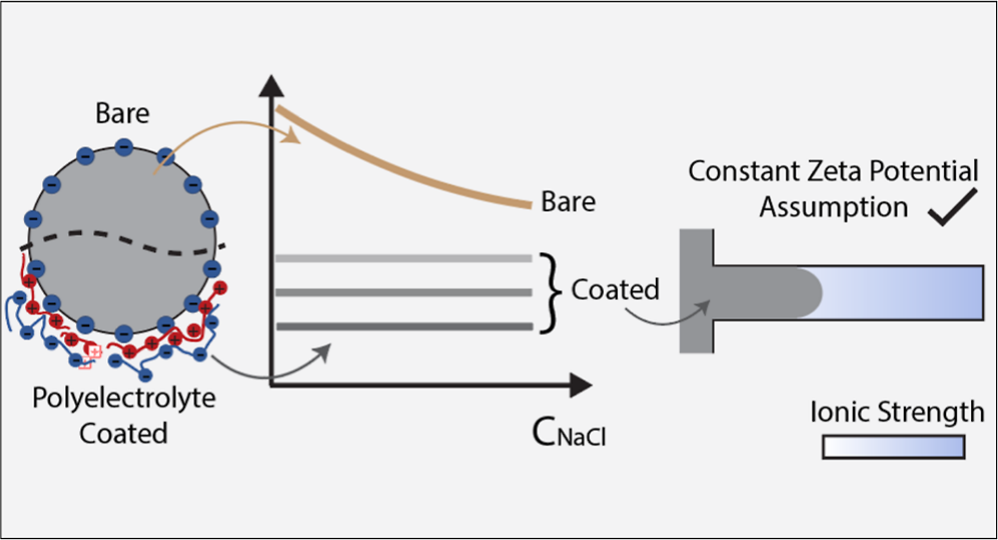

Diffusiophoresis, the movement of particles under a solute concentration gradient, has practical implications in a number of applications, such as particle sorting, focusing, and sensing. For diffusiophoresis in an electrolyte solution, the particle velocity is described by the electrolyte relative concentration gradient and the diffusiophoretic mobility of the particle. The electrolyte concentration, which typically varies throughout the system in space and time, can also influence the zeta potential of particles in space and time. This variation affects the diffusiophoretic behavior, especially when the zeta potential is highly dependent on the electrolyte concentration. In this work, we show that adsorbing a single bilayer (or 4 bilayers) of a polyelectrolyte pair (PDADMAC/PSS) on the surface of microparticles resulted in effectively constant zeta potential values with respect to salt concentration throughout the experimental range of salt concentrations. This allowed a constant potential model for diffusiophoretic transport to describe the experimental observations, which was not the case for uncoated particles in the same electrolyte system. This work highlights the use of simple polyelectrolyte pairs to tune the zeta potential and maintain constant values for precise control of diffusiophoretic transport.

## 1 Introduction

Diffusiophoresis is a nonequilibrium process which was first described by Derjaguin and co-workers.^1,2^ A solute concentration gradient can lead to a particle diffusiophoretic movement. This gradient can be any form (an electrolyte^3^ or a nonelectrolyte^4^). The diffusiophoretic velocity depends on the interaction strength between the solute molecules and the particle surface. In electrolytes, the driving force is a relative solute concentration gradient. The mobility depends on the zeta potential of the particle (ζ_p_) and the diffusivity contrast between the cation and anion [β = (*D*_+_ – *D*_–_)/(*D*_+_ + *D*_–_)] when the Debye layer (κ^–1^) is relatively small compared to the particle radius (*a*).^5^ Theoretical aspects of charged particle diffusiophoresis with different conditions, such as thin, arbitrary, or thick Debye layers, have all been explored previously.^6^ Diffusiophoresis and osmosis have practical implications in various areas, including particle sorting,^7^ particle focusing,^8,9^ surface characterization,^10^ and nanopore DNA sensing,^11^ among others.^12,13^

Zeta potential is influenced by the ionic strength or ion type^14^ and any changes may influence the diffusiophoretic mobility. For example, theoretical calculations (based on eq 2) show that a change of zeta potential from −10 to −55 mV results in approximately an order of magnitude difference in diffusiophoretic velocity for the same NaCl gradient. Previously, we experimentally demonstrated that the change in salt concentrations influences the diffusiophoretic particle mobility, resulting in a deviation between numerical predictions based on constant zeta-potential and experimental results for the case when the particle zeta potential strongly depends on the salt concentration.^15^ We were able to achieve good agreement between experiments and simulations by adjusting the zeta potential according to the salt concentration at a given location and time. Other electrokinetic surface models such as constant surface charge^16^ or charge regulation models,^17,18^ can be used to obtain different diffusiophoretic mobility expressions. Recently, Lee et al.^19^ provided a guideline for selecting the most appropriate model (constant zeta potential, constant surface charge, or charge-regulation) for a given diffusiophoretic system. In this work, we study the effect of coating particles with polyelectrolytes in a layer-by-layer fashion. Polyelectrolyte-coated particles can possess effectively constant zeta potential^20^ over typical salt concentration ranges for diffusiophoretic experiments. It has been previously shown that stable zeta potential values of particles at high ionic concentrations (until 200 mM NaCl) can be achieved upon coating multiple layers of polyelectrolytes.^20^ Moreover, nonuniformities in surface charge can also be reduced by coating with polyelectrolytes.^21^ Thus, there are clear benefits to coating particles with polyelectrolytes.

Layer-by-layer adsorption is a relatively simple technique based on consecutively adsorbing anionic and cationic polyelectrolytes onto a surface, thereby forming functional thin films.^22^ The polyelectrolyte adsorption mechanism is governed by interactions between the surface and polyelectrolyte. For oppositely charged polyelectrolytes and particles, polymer chains are attracted to the oppositely charged surface, mainly by electrostatic interactions. It was also shown that non-Coulombic forces (such as hydrophobic interactions or hydrogen bonding) play a role in the adsorption process.^23^ Overall, the layer assembly process is entropically driven by releasing counterions upon polyelectrolyte adsorption.^24^

The adsorption process of polyelectrolytes is influenced by various conditions such as the polyelectrolyte concentration,^25−27^ molecular weight,^28^ ionic strength of the solution,^25,28−33^ ion type,^34^ and pH^26,29,30^ of the solution. These parameters affect the adsorbed amount, as well as the adsorption kinetics. The ionic strength of the solution is an important parameter as it controls the entropic gain.^35^ The terms intrinsic and extrinsic charge compensation distinguish the charge balancing mechanism, whether the polyelectrolyte charge is balanced with the oppositely charged polyelectrolyte (intrinsic) or with counterions in the solution (extrinsic).^36^ At high salt concentrations, the extrinsic charge compensation is more significant and changes the layer properties, such as layer thickness and charge. These properties can be modified by adjusting the salt concentration during coating.

This study aims to explore experimentally the effect of adsorbed polyelectrolyte pairs on particle diffusiophoresis. We show the effect of the salt concentration used during coating, the particle’s initial surface charge, and the number of (bi)layers on the resulting particle zeta potential behavior. After adsorbing polyelectrolytes on the polystyrene (PS) particles under various conditions, we tested the coated particles further in diffusiophoretic experiments using a dead-end channel microfluidic system. We also performed simulations by solving unsteady Stokes and convection-diffusion equations in 3-D to predict the behavior of the particles and compared these to our experimental observations. For polyelectrolyte-coated particles, diffusiophoretic migration of particles could be accurately described using a constant zeta potential in contrast to uncoated particles. We also highlight the versatility of polyelectrolyte coatings for diffusiophoretic experiments, as resulting constant zeta potential values can be tuned based on the coating conditions.

## 2 Theoretical Background and Simulations

### 2.1 Theoretical Diffusiophoretic Movement

The direction and speed of the particle diffusiophoresis are determined by the interaction strength between the solute and the particle. Mathematically, the diffusiophoretic velocity for rigid particles^3,5^ reads as
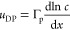
1where Γ_p_ is the diffusiophoretic mobility which determines the interaction strength between solute and particle surface. That can be analytically found under certain assumptions. When the Debye length (κ^–1^) is much smaller than the particle radius (κ*a* → ∞), the equation for diffusiophoretic mobility^3^ reads as
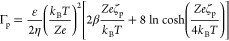
2where ε is the medium permittivity, η is the medium viscosity, *k*_B_ is the Boltzmann constant, *T* is the medium absolute temperature, *e* is the elementary charge, and *Z* is the valence of the solute (*Z* = *Z*_Na_^+^ = −*Z*_Cl_^–^ = 1). The first term quantifies the electrophoretic contribution. A spontaneous electric field is built up, which creates an electrostatic force on the particle due to the ions diffusivity contrast β = (*D*_+_ – *D*_–_)/(*D*_+_ + *D*_–_), where *D*_+_ = 1.33 × 10^–9^ m^2^/s for Na^+^ and *D*_–_ = 2.03 × 10^–9^ m^2^/s for Cl^–^ at room temperature (β = −0.208). The second term describes the chemiphoretic contribution. This contribution is due to the nonuniform adsorption of counterions due to the concentration gradient, which results in an osmotic pressure difference that drives the particle.^3^

The particle diffusiophoretic mobility expression is altered when the particle size is comparable with or smaller than the Debye length (κ*a* ≤ 1).^3,37,38^ In our work, the Debye length (≈5–15 nm, based on the front particle location salt concentration, given in Akdeniz et al.^15^) is 35–100 times smaller than the particle radius (≈500 nm).

The particle diffusiophoresis velocity description might change from the above rigid particle explanation when it has a coated layer.^39−45^ This deviation depends on the properties of the adsorbed layer,^44^ such as layer thickness (*d*), flow penetration—Brinkman parameter λ^–1^, the layer charge density (*N*), and surface charge density σ. However, due to the relatively small thickness of the coating layer^46−48^ compared to the particle size, the diffusiophoretic expression for a rigid noncomposite particle can be applied for the case of a single bilayer (or 4 bilayers)-coated particle, as we will demonstrate later on. Alternative diffusiophoretic expressions would be required when the particle size is compatible with polyelectrolyte layer thicknesses, as could be the case when using nm-scale particles. Suitable expressions for this case can be found in.^44,45^

The particle zeta potential value is needed to solve equation eq 2. We estimated the zeta potential value from the electrophoretic mobility (see Characterization). However, similarly to the diffusiophoretic case given above, electrophoretic mobility might be influenced by the polyelectrolyte layer (see core–shell discussion^49^), and zeta potential started to lose its meaning.^50^ However, again we are in the limit that the particle is quite large compared to the polyelectrolyte thickness. In this limit, the soft particle description approaches the hard models.^49^ Therefore, we assumed particles to be rigid and showed zeta potential values in the paper.

### 2.2 Simulations

Simulations are performed in a similar manner to our previous work in COMSOL Multiphysics 6.0 (see our previous work for additional details).^15^ COMSOL Multiphysics 6.0 was used to solve the equations using the finite element method in a time-dependent manner. P2 + P1 elements (second-order elements for velocity and first-order elements for pressure) were used to solve the Stokes and continuity equations. The mass transport equation is solved using second-order Lagrange elements to compute the concentration field. Mesh independence was assessed through successive mesh refinements by examining the concentration and velocity profiles.

#### 2.2.1 Equations with Boundary and Initial Conditions

Unsteady Stokes (eq 3) and fluid continuity (eq 4) equations for incompressible fluids were used to describe the fluid flow inside and outside of the dead-end channel.
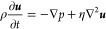
3
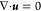
4where ρ is the fluid density, η is the fluid viscosity, ***u*** is the fluid velocity vector, and *p* is the pressure.

**Figure 1:**
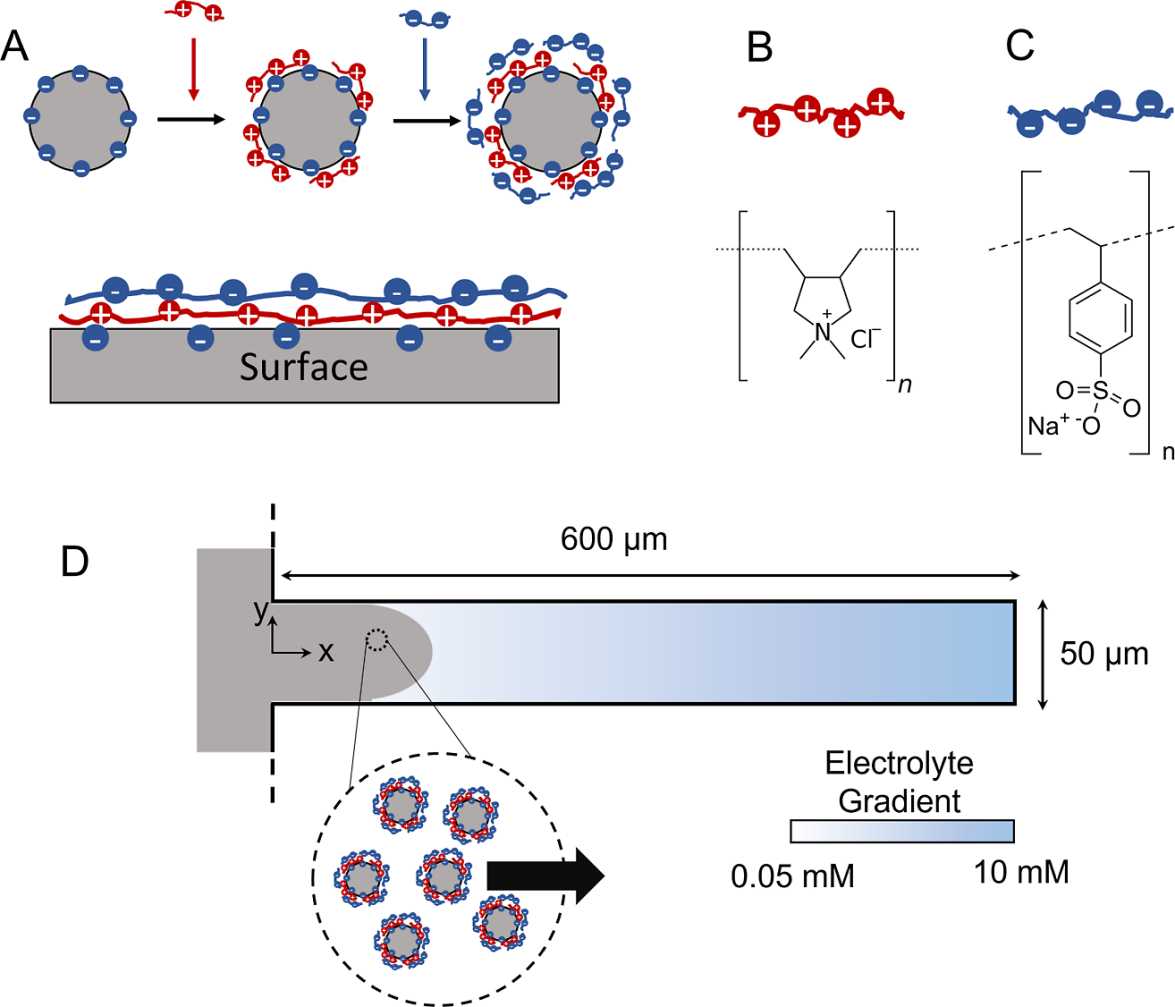
Schematic of the polyelectrolyte adsorption on the particle surface and the experimental system. (A) Layer-by-layer polyelectrolyte adsorption on the particle surface. The particles, which have a negative charge, are mixed with a polyanion and a polycation. The chemical structure of poly(diallyldimethylammonium chloride)—PDADMAC (B), and poly(sodium 4-styrenesulfonate)—PSS (C) are also shown. (D) Once the polyelectrolytes have been adsorbed onto the particles, they are used in a dead-end channel experiment to observe their diffusiophoretic behavior.

All walls are assumed to be impermeable to the solution (water) or solute (salt). The main channel has an inlet and an outlet, where the inlet velocity was set as 280 μm/s (*y* direction in Figure 1). The pressure at the outlet boundary is set to 0 Pa as an arbitrary value, as only the pressure gradient matters for incompressible flow. We defined an effective wall slip velocity given by the diffusio-osmotic velocity at all dead-end channel walls
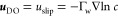
5

The magnitude of the diffusio-osmotic mobility (Γ_w_) is equal to the magnitude of the diffusiophoretic mobility (Γ_p_ given in eq 2) when the Debye length is negligibly small compared to the particle radius (κ*a* → ∞). Thus, the diffusio-osmotic and diffusiophoretic velocities are equal to each other in magnitude, but they are in opposite directions (***u***_DO_ = −***u***_DP_).^3^ For the zeta potential of the wall (PDMS), we have used the ζ = *a* + *b* log_10_(*c*_*i*_) equation (where *a* = 6.27 mM, *b* = 29.75 mV, and *c*_*i*_ is the salt concentration). Previously, we showed the agreement between this equation and the streaming potential measurements. (Please refer to Figure S3 of Akdeniz et al.^15^).

The solute concentration distribution was estimated by solving the transient convection–diffusion equation.
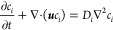
6where *D*_*i*_ is the ambipolar diffusion coefficient *D*_*i*_ = 2*D*_+_*D*_–_/(*D*_+_ + *D*_–_) (where *D*_NaCl_ = 1.61 × 10^–9^ m^2^/s at room temperature). The initial concentration inside the dead-end channel was 10 mM NaCl. The main channel and its inlet concentration are set to 0.05 mM NaCl to match experimental conditions.

The particle dynamics were also calculated using transient convection–diffusion equation as in previous studies.^15,16,38^ This continuum approach accounts for particle diffusion (Brownian motion) and fluid convection, treating particles as point sources, neglecting particle–particle and particle–wall interactions for simplicity. The convective term includes the diffusiophoretic velocity of the particles combined with the fluid flow generated by diffusio-osmosis within the dead-end channel.
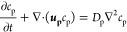
7where *D*_p_ is the particle diffusion coefficient, estimated by the Stokes–Einstein equation (*D*_p_ = *k*_B_*T*/6πη*a*). ***u***_**p**_ was determined as the sum of particle diffusiophoresis (***u***_**DP**_ = Γ_p_∇ ln *c* from eqs 1 and 2) and the fluid flow (***u*** from eqs 3 and 4). The initial concentration value of particles inside the dead-end channel was set to 0 while for the main channel and the inlet, the particle concentration was set to 1 as we are interested in tracking a particle-front in the dead-end channel. For presenting the simulation results of the penetration depth value, we arbitrarily chose the particle concentration threshold *c*_p_ 0.1 in the text. The effect of the higher threshold is analyzed in Figure S4 in the Supporting Information.

## 3 Experimental Section

### 3.1 Materials

Poly(diallyldimethylammonium chloride) (PDADMAC, 30 wt % in water, *M*_w_ ≈ 200–350 kDa) solution and poly(sodium 4-styrenesulfonate) (PSS, 25 wt % in water, *M*_w_ ≈ 200 kDa) were purchased from Merck (The Netherlands). Polyelectrolyte solutions were used directly without further purification. RTV-615 A (Permacol B.V, Ede, The Netherlands), prepolymer (1020 kg/m^3^) and RTV-615 B (Permacol B.V, Ede, The Netherlands), and curing agent (990 kg/m^3^) were used to produce polydimethylsiloxane (PDMS). Sodium chloride (NaCl) (99.96%) was obtained from AkzoNobel (The Netherlands). PS-FluoRed-1.0; 1.14 μm (2.5 wt %, SD = 0.03 μm, abs/em = 530/607 nm) and PS-RhB-PEG-Fi70–1: 1.09 μm (2.5 wt %, SD = 0.04 μm, abs/em = 560/584 nm) were obtained from Microparticles GMBH (Berlin, Germany). FluoSpheres PS-carboxylate; 1.00 μm (2 wt %, SD = 0.02 μm, abs/em = 580/605 nm) was obtained from Thermo Fisher (United States).

### 3.2 Device Fabrication

The device production is similar to our previous article.^15^ To summarize, PDMS is prepared by mixing the prepolymer (RTV-615 A) and the curing agent (RTV-615 B) with a 10:1.5 ratio. The prepolymer and the curing agent were blended for at least 5 min to obtain a uniform mixture. After the mixture was placed in a desiccator to degas for at least half an hour, it was poured onto the two Si-wafer molds (Si-wafer without any structure (flat) and Si-wafer with positive dead-end channel structure) and degassed again to remove all bubbles. The PDMS mixture was cured for 4 h at 80 °C in an oven. After activating the flat and structured PDMS surfaces using a Femto plasma cleaner (Diener electronic GmbH, Ebhausen, Germany), O_2_ plasma, for 12 s at 100 W, they were bounded to each other. Prepared microfluidic devices were then soaked under deionized water (Milli-Q) before performing an experiment to reduce the water permeation through the PDMS walls.^51^

The microfluidic device overall contains a main channel connected to dead-end channels. The main channel is 600 μm wide and 100 μm high. The dead-end channel is 50 μm wide (W), 10 μm high (H), and 600 μm long (see 2D schematic in Figure 1D). The uncertainty of these dimensions, based on SEM, is around 1 μm. For the picture and cartoon of the experimental system, please refer to Supporting Information S1.

### 3.3 Particle Coating

0.1 g/L polyelectrolyte aqueous solutions (0.1 g/L) were prepared in 250 mL glass flasks containing 0, 3, 5, 10, 25, and 50 mM electrolyte concentrations (NaCl). 125 μL of particle suspension (2%) was mixed with 10 mL of polycation solution (PDADMAC) in a 15 mL polypropylene conical centrifuge tube. This suspension was mixed with a vortex mixer for at least 5 min. The suspension was then sonicated for 25 min in ElmaSonic P (Elma Schmidbauer GmbH, Singen, Germany). After sonication, a centrifuge (Corning LSE, New York, USA) was used to separate the particles from the polyelectrolyte solution. The sample was centrifuged at 6000 rpm for around 30 min to obtain a particle-free supernatant that contains unadsorbed polycation (PDADMAC). The ∼9.5 mL supernatant was removed from the centrifugal tube, and the remaining mixture was washed with ∼9.5 mL of the same salt concentration that was inside the polyelectrolyte solution. 2 μL of the particle suspension was mixed with 5 mM NaCl solution for the zeta potential analysis to ensure dilute behavior. The coating process was continued with the remaining (∼0.5 mL) particle suspension by adding polyanion solution. 10 mL of polyanion (PSS) solution with 0.1 g/L was added to the particle suspension, and the same process was repeated. This time, after centrifugation to obtain particle-free supernatant that contained unabsorbed polycation solutions, the supernatant was collected for UV–vis analysis to determine the PSS concentration. The calibration curve used to determine the PSS concentration can be found in Supporting Information S2. The coating process with a polycation and polyanion was repeated until the desired layer was obtained. After coating with the desired layer of polyelectrolytes, the particle suspension was washed with Milli-Q water at least three times prior to diffusiophoretic experiments to remove any residual salts.

### 3.4 Characterization

The structure and morphology of the bare and coated particles were examined using scanning electron microscopy (SEM) (JSM-6010LA, JEOL, Japan). The particle samples for SEM were first placed on small glass slides. Then, the samples were placed in a vacuum overnight before sputtering. A 5 nm thin Pd/Pt alloy layer was sputtered on particles using a Quorum Q150T ES (Quorum Technologies Ltd., UK), and the particles were analyzed.

We determined the zeta potential of the particles by measuring the electrophoretic mobility of the particles. Coated (or uncoated) particle suspension was mixed with 1, 3, 5, 7, and 10 mM NaCl solutions (obtaining ≈0.005% w/v particle concentrations), and the electrophoretic mobility of particles was then measured at these salt concentrations. A Zetasizer Nano-ZS (Malvern Panalytical B. V., Almelo, The Netherlands) device was used to determine the electrophoretic mobilities, which is correlated with the zeta potential ***U***_electrophoresis_ = 2εε_0_ζ*f*(κ*a*)***E***/(3η). Henry’s function *f*(κ*a*) was estimated by Swan’s approach for each case.^52^

Streaming potential measurements for the PDMS flat sheets were performed with an Electrokinetic Analyzer, SurPass I (Anton Paar, Graz, Austria),^15^ and zeta potential estimated via the Helmholtz–Smoluchowski equation. Agreement over the experimental salt concentration range was observed with the values in Kirby and Hasselbrink.^53^ To describe the zeta potential change with salt concentration for the PDMS surface, we used the following equation: ζ = *a* + *b* log_10_(*c*) with *a* = 6.27 mV and *b* = 29.75 mV where *c* is in M, as in our previous work.^15^

UV–vis spectroscopy was used to analyze the poly(styrenesulfonate) amount,^22^ using a UV–vis spectrophotometer (Shimadzu UV-1800, Japan) at λ_max_ = 225 nm, the maximum absorbance wavelength for PSS. See Supporting Information S2 for the details of the analysis and calibration curves.

### 3.5 Diffusiophoretic Experimental Protocol

A plastic syringe was used to fill the dead-end channel with a 10 mM NaCl solution. Then, an air bubble was passed through the main channel. Meanwhile, the particle suspension was sonicated for at least 5 min. Then, the particle suspension was passed through using a syringe pump (Harvard Apparatus, PHD-Ultra, Massachusetts, USA). An inverted microscope (Zeiss Axio Observer Z1, Carl-Zeiss, Jena, Germany) was employed with a 20× *f*/0.4 objective (depth of field is 5.8 μm, Zeiss LD Plan-Neofluar, Carl-Zeiss) to visualize the particle movement inside the dead-end channel. The particle motion was captured by a CCD camera (Hamamatsu, Japan) with a 2048 × 700 pixels resolution. The images are sequentially captured for 5 min in 10 frames-per-second (fps). The set of microscope images was analyzed in ImageJ, an open-source image analysis software^54^ to determine the penetration depth of particles into the dead-end channel.

## 4 Results and Discussion

### 4.1 Polyelectrolyte Adsorption

The schematic of the layer-by-layer polyelectrolyte adsorption on a particle surface is shown in Figure 1A. Negatively charged particles interact with the oppositely charged polyelectrolytes (polycations) in solution by mainly electrostatic interactions. Here, we used PS-carboxylate as the negatively charged particle and a 0.1 g/L PDADMAC solution (Figure 1B) as the polycation in 25 mM NaCl. We determined the zeta potential value at each stage to characterize the surface charge.^27,55^ The zeta potential of the bare PS-carboxylate particles was determined as −68.1 ± 0.6 mV in 5 mM NaCl from electrophoresis measurements. Once the polyelectrolyte is adsorbed on the particle surface, depending on the polyelectrolyte amount,^25^ the particle surface charge becomes less negative in magnitude or switches to a positive value. In our case, the zeta potential became +47.3 ± 4.3 mV after coating with the polycation. This is due to the charge overcompensation and resulting charge inversion.^56^ The process can be continued with polyanion adsorption (PSS, Figure 1C). After the adsorption of the polyanion (0.1 g/L PSS in 25 mM NaCl solution), the sign of the particle zeta potential flips again and becomes a negative value: −64.9 ± 1.8 mV.

**Figure 2:**
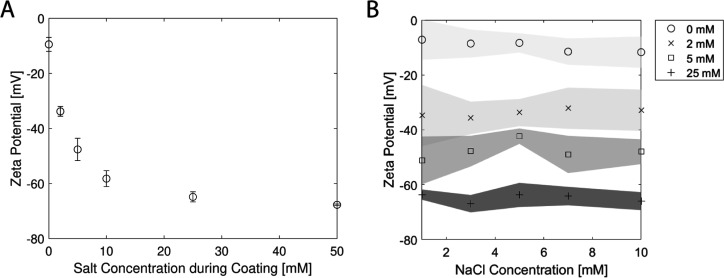
Impact of electrolyte concentration on polyelectrolyte adsorption. (A) The averaged zeta potential values are plotted against the electrolyte concentration in the coating solution. The error bars represent the 95% confidence interval of the values at 1–10 mM NaCl. (B) The zeta potential of the coated particles with respect to NaCl concentration in solution during zeta potential measurement, showing near-constant values. The legend indicates the electrolyte concentrations that were used in the coating process. Other electrolyte concentrations are provided in Supporting Information S3. The shadow areas represent the 95% confidence interval of three or four separate coating experiments.

Factors such as solution pH, polyelectrolyte molecular weight, contact time, temperature, and the ionic strength in the coating solution affect the adsorption process and thus the resulting surface charge.^25,26,28−33^ Here, we have explored the effect of salt concentration during coating while keeping other parameters constant, and we determined the zeta potentials of coated particles. Figure 2A indicates the averaged zeta potential values of bilayer-coated particles for varying electrolyte concentrations used in the particle coating step. The figure shows that particles have higher absolute values of zeta potential with the same bilayer (PDADMAC/PSS) when coated in higher salt concentrations. A similar observation was shown for single PEI and PSS adsorption, where particles have higher electrophoretic mobility when the salt concentration is higher.^57^ The change in zeta potential value is related to the surface charge density of the final layer (PSS), related to the structural change of the polyelectrolyte at the particle surface and the adsorbed amount.^25^ The polyelectrolyte molecules form a coiled-like structure at high salt concentrations and a more rod-like shape or extended structure at low concentrations. This structural change in the polyelectrolyte molecule is associated with a change in the electrostatic interaction between charged monomers.^58^ When a polyanion contacts a polycation, it forms a polyelectrolyte complex, mainly driven by entropic gain due to the release of counterions. The salt concentration affects the electrostatic interaction between the polyanion and the polycation. Adding salt in bulk reduces the electrostatic interaction between the opposite polymer segments since the charge starts to be compensated extrinsically by the salt ions present in the solution.^59^ At high electrolyte concentrations, the PSS forms a coiled-like structure and creates more loops and tails at the surface, where complexes with PDADMAC are mostly intrinsically compensated. This leads to the available excess charge on the structure, which translates into high surface charge density and thus high absolute zeta potential values.

We estimated the PSS adsorption amount at the particle surface using the supernatant concentration value after coating the particles with 0.1 g/L PSS (the polymer dose used is 40 mg of PSS/g of the particle). We found ∼0.2 mg/m^2^ PSS amount adsorbed by the particle surface. That is consistent with the value found in the literature.^25^ We determined it for different salt concentrations, and the difference is not significant at 95% confidence. See Supporting Information S2 for more information about adsorption value.

After adsorbing a bilayer of PDADMAC/PSS on PS-carboxylate particles at different salt concentrations, we checked the zeta potential values of the coated particles at different salt concentrations (Figure 2B). We found that the zeta potential of the coated particles is quite stable throughout the salt concentration range of 1–10 mM NaCl. The stable potential result can be explained due to structural changes at the polyelectrolyte layer. Increasing the bulk salt concentration leads to a reorientation of the polymer layers (swelling or shrinking of the polymer layer),^50,60,61^ which is further related to the charge screening.^20^ It is important to note that the absolute value of the zeta potential started to decrease at an electrolyte concentration above 25 mM NaCl. Coating a higher number of polyelectrolyte bilayers results in a stable zeta potential over the 200 mM NaCl range.^20^

### 4.2 Diffusiophoretic Behavior of Polyelectrolyte-Coated Particles

We tested our coated particles in a microfluidic device containing dead-end channels (Figure 1D) where particle diffusiophoresis has been previously analyzed.^10,15,16,38,62,63^ Particles can enter the dead-end channel when an electrolyte gradient is present inside the dead-end channel when β and ζ_p_ are <0. The electrolyte gradient (in the *x* direction) leads to particle diffusiophoresis and channel wall diffusio-osmotic flow. Inside the dead-end channel, the particle velocity depends on the diffusiophoretic velocity (***u***_**DP**_) and the fluid flow (***u***). Particle diffusiophoresis is quantified by the relative gradient and a mobility term (eq 2). The generated osmotic flow creates a recirculating flow inside the dead-end channel, toward the main channel near the wall, and toward the dead-end near the center (see the Supporting Information in our previous work^15^).

Particle diffusiophoresis and wall diffusio-osmosis depend on the relative electrolyte gradient and mobility term (eq 1). The mobility is determined by the strength of the interaction between the corresponding surface and the electrolyte present in the solution. Mathematically, diffusiophoresis and the diffusio-osmotic velocity have the same magnitude, but an opposite sign (***u***_**DP**_ = −***u***_**DO**_)^5^ when the Debye length is much smaller than the particle radius. The zeta potential value of the particle or wall and diffusivity contrast (β) determine the flow direction and magnitude. It was previously observed that the electrolyte concentration inside the dead-end channel can influence the zeta potential of the particles, which further affects the dynamics of the particles.^15,19^ Here, we observed stable zeta potential values throughout the experimental range after coating with polyelectrolytes, allowing for experimental investigation with a constant zeta potential of particles.

**Figure 3:**
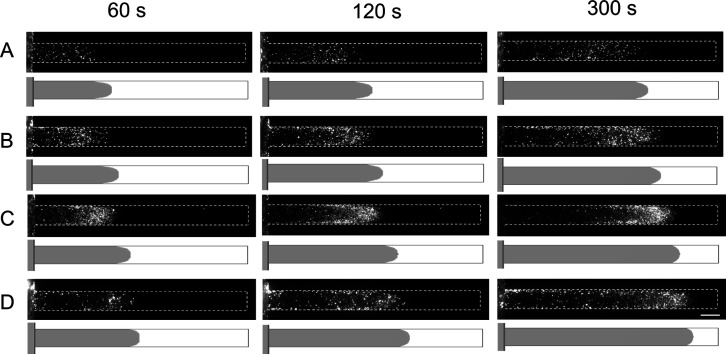
Results of the diffusiophoresis experiments with corresponding numerical predictions for times: *t* = 60, 120, and 300 s. All of the diffusiophoresis experiments were conducted with a 10 mM NaCl concentration in the dead-end channel, while the main channel contained 0.05 mM NaCl concentrations with polyelectrolyte adsorbed particles. The particles in the main channel were coated with PDADMAC/PSS (1 BL) with (A) 2 mM NaCl, (B) 5 mM NaCl, (C) 10 mM NaCl, and (D) 25 mM NaCl electrolyte concentrations. The gray area in numerical prediction, which is located below each experimental observation, represents the particle concentration >0.1. In the numerical prediction, the wall zeta potential varies with the electrolyte concentration, while the particle zeta potential is kept constant at (A) −35, (B) −45, (C) −60, and (D) −65 mV. Scale bar = 50 μm.

Diffusiophoresis experiments were performed with one bilayer of PDADMAC/PSS-coated particles that were coated at varying salt concentrations during coating. The experimental results for coated particles, at indicated salt concentrations during coating, are shown for the corresponding times of t = 60, 120, and 300 s (Figure 3). The particles penetrate more into the dead-end channel when their absolute zeta potential values are higher. The higher absolute zeta potential value leads to a higher diffusiophoretic velocity for β < 0 electrolytes, since the resulting phoretic mobility is higher. The diffusiophoretic mobility is 2.36 × 10^–10^ m^2^/s for −35 mV zeta potential particles and 5.92 × 10^–10^ m^2^/s for −65 mV particles. For this reason, Shin et al.^10^ showed that diffusiophoresis experiments in the dead-end channels could be used for low-cost zeta potentiometry. It is important to emphasize that estimating the average zeta potential in this manner does not necessarily give the exact penetration of the particles, since the zeta potential can change substantially with the electrolyte concentration in the dead-end channel.^15,19^

We simulated the diffusiophoretic particle behavior using a 3-D model by solving the unsteady Stokes equation and the convection–diffusion equation for both the salt (NaCl) and the particles (see the Simulation section). In the simulation, the diffusiophoresis expression for a charged rigid particle was used.^3,5^ In addition, the zeta potential of the particles was kept constant at the values given in Figure 2A. The zeta potential of the wall (PDMS) is adjusted for the local electrolyte concentration in the simulations since the zeta potential of PDMS is highly dependent on the electrolyte concentration, especially at low concentrations (<10 mM).^15,53^ The influence of the wall-generated osmosis is dominant when the wall zeta potential is higher than the particle (ζ_p_ ≪ ζ_w_).^62^ The gray area shows the possible particle positions inside the dead-end channel from the numerical prediction where *c*_p_ > 0.1 (Figure 3). Figure 3 shows that the numerical predictions with constant zeta potential values for the particles agree with the experimental observations for 1 BL polyelectrolyte-coated particles.

**Figure 4:**
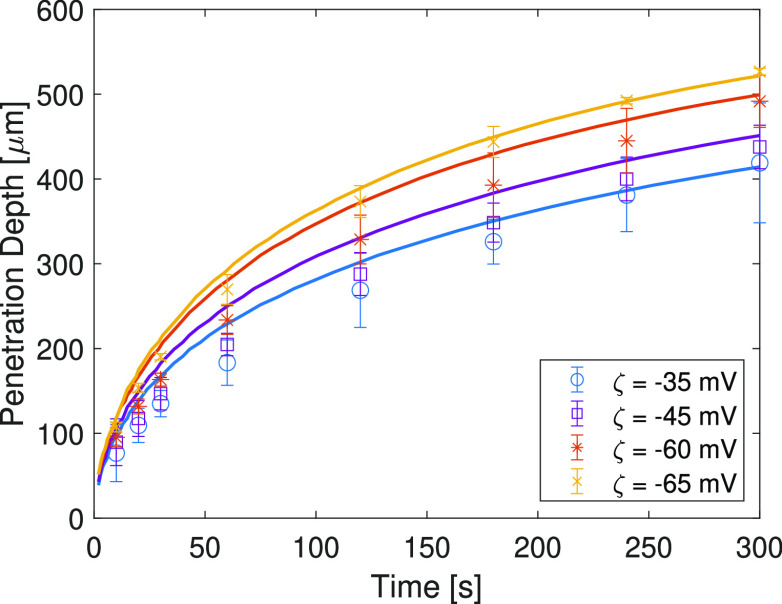
Temporal position of the leading particle (referred to as penetration depth) is experimentally measured and numerically predicted. The legend indicates the particle zeta potential values used in the simulations. The particles were coated with 0.1 g/L PDADMAC/PSS pair, and salt (NaCl) concentrations during coating were 2, 5, 10, and 25 mM, resulting in zeta potential values of −35, −45, −60, and −65 mV respectively. The error bar represents the 95% confidence interval based on at least three measurements. The solid line represents the simulation results where the particle concentration is 0.1.

We qualitatively analyzed the experimental and numerical predictions by determining the penetration depth (Δ*x*) in time, which corresponds to the leading particle position. The results for all coated particles are shown in Figure 4. The solid lines represent the numerical prediction of the leading particle position (where the particle concentration is 0.1), and the markers show the experimental observations. Figure 4 shows that the numerical prediction for longer times (>180 s) aligns with the experimental observations. The numerical prediction somewhat overestimates the penetration depth, especially for the early stages and low zeta potential values. This overestimation may result from the arbitrarily chosen threshold value *c*_p_ > 0.1, as more similar values were obtained when selecting higher threshold values for *c*_p_ (see Figure S4 in the Supporting Information).

An effective diffusion coefficient (*D*_eff_) can be extracted using the penetration depth versus time of the particles by fitting with .^64^ The values of the fitted parameters are given in Table 1, and fitting details are given in Supporting Information S5. The values are approximately 3 orders of magnitude larger than the particle diffusivity value according to the Stokes–Einstein equation (*D*_p_ = *k*_B_*T*/(6*πηa*) = 4.3 × 10^–13^ m^2^/s), while they are less than an order of magnitude smaller than the monovalent ion diffusivity (1–2 × 10^–9^ m^2^/s). This result underlines the relevance and power of the diffusiophoretic processes, especially in the presence of aqueous electrolyte concentration gradients. The calculated effective particle diffusion coefficients are of the same order of magnitude as the diffusiophoretic mobilities, which are also given in Table 1. However, it is difficult to relate these effective diffusion values directly to the diffusiophoretic mobilities in the presence of wall diffusio-osmosis. Unlike the coflow system,^65^ the particles experience both the diffusiophoretic motion and the fluid flow generated by the diffusio-osmosis at the PDMS surface, which varies over the height and width of the dead-end channel. The effective diffusion coefficient value is higher than the diffusiophoretic mobility at low zeta potential values, and it is lower at other zeta potential values (Table 1). We have also found *D*_eff_ values for numerical calculations where the particle concentration is >0.1 and 0.5. The results are shown in Table S1. The values are between the experimental values and the fitted curves in Figure S5.

**Table 1:** Effective Diffusion Coefficient of the 1 BL Polyelectrolyte-Coated Particles^a^

*c*_salt_ [mM]	ζ_avg_ [mV]	*D*_eff_ × 10^10^ [m^2^/s]	Γ_p_ × 10^10^ [m^2^/s]
2	–33.8 ± 1.8	2.97 ± 0.12	2.27
5	–47.6 ± 4.0	3.30 ± 0.10	3.75
10	–58.2 ± 2.9	4.15 ± 0.11	4.85
25	–64.9 ± 1.8	5.01 ± 0.19	5.92

a The salt concentration during coating (*c*_salt_) is given with the representative averaged zeta potential values (ζ_avg_). The fitting procedure is given in Supporting Information S5. The values after ± are the 95% confidence level of the fit.

In addition to the observation described above, we did not observe any change in the particle fluorescence after coating with 1 BL of PDADMAC/PSS, as can be seen in Figure 3. We also explored diffusiophoresis of polycation-coated particles (leaving the particle with a positively charged surface). These particles interact electrostatically with the negatively charged wall and get stuck.

### 4.3 Multilayer Polyelectrolyte Coating

To study the effect of additional coating layers, we adsorbed PDADMAC/PSS on the particles by the layer-by-layer method with up to 10 BLs. However, the chance of particles to aggregate increases since PDADMAC functions as a coagulant.^25,66^ Previous studies using PAH/PSS as opposed to our PDADMAC/PSS showed that the percentage of singlets, doublets, triplets, or higher-order aggregates did not change with up to 14 deposited polyelectrolyte layers.^47^

**Figure 5:**
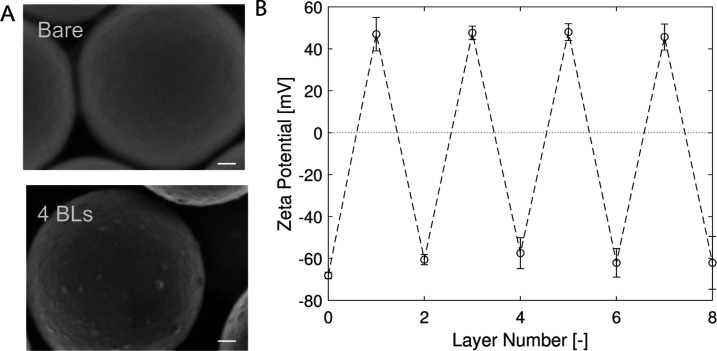
Characterization of particles coated with polyelectrolyte multilayers. (A) Scanning electron microscope images of the bare and 4 BLs of PDADMAC/PSS (25 mM NaCl)-coated particles are given. Scale bar = 100 nm. (B) Zeta potential values of coated particles (in 5 mM NaCl solution) at each stage of the coating procedure. The error bar given in the figure shows the 95% confidence interval of three coating experiments.

We have extended our observations from 1 bilayer to 4 bilayer polyelectrolyte adsorption. We filtered the suspension with a 5 μm porous filter prior to the diffusiophoresis experiments to remove the already-formed aggregates. The adsorbed polyelectrolyte multilayer on the particle surface is shown in Figure 5A. The scanning electron microscope (SEM) image of the particle surface indicates that the particle surface became rougher after coating with polyelectrolytes, as the thickness of the layer and roughness increases with layer number consistent with previous reports.^59,67^

The zeta potential analysis (Figure 5) indicates that the particle zeta potential alternates between positive and negative values, depending on the final group at the surface (PDADMAC or PSS). This change is typical for multilayer systems.^47,48^ This indicates that the polyelectrolyte is adsorbed in each stage.

**Figure 6:**
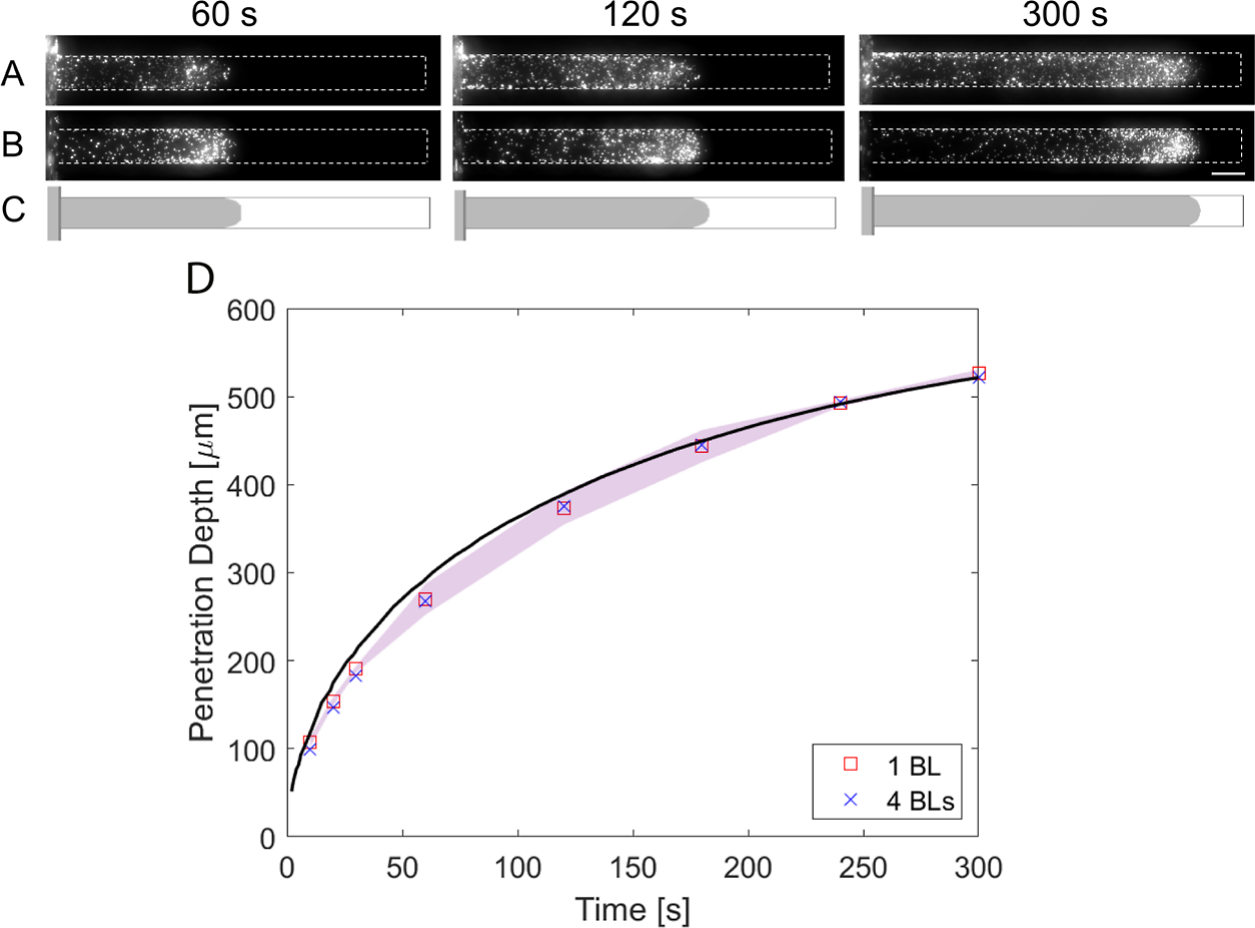
Results of the diffusiophoretic experiments with corresponding numerical predictions for times: *t* = 60, 120, and 300 s. The diffusiophoretic experiments were conducted with a 10 mM NaCl concentration in the dead-end channel, while the main channel contained 0.05 mM NaCl concentrations with polyelectrolyte-coated particles. The particles in the main channel were coated with (A) 1 BL and (B) 4 BLs of PDADMAC/PSS (in 25 mM NaCl). Scale bar = 50 μm. (C) The gray area in the simulation represents the region with particle concentration >0.1. In the numerical simulations, the wall zeta potential is a function of the electrolyte concentration, whereas the particle zeta potential is kept constant at −65 mV to match the experimental conditions. (D) The particle penetration depth is plotted against time. The shadow area represents the 95% confidence interval of at least three measurements. The solid line represents the simulation where particle concentration is >0.1.

After the characterization of the particles, we performed diffusiophoretic experiments to test the possible change in their diffusiophoretic motion. In these diffusiophoretic experiments, similar to those described above, the dead-end channel is filled with 10 mM NaCl. We compared the results of the diffusiophoresis experiments between 1 BL and 4 BLs coated particles (0.1 g/L of PDADMAC/PSS in 25 mM NaCl) in Figure 6, which shows the microscope images of diffusiophoretic experiments at 60, 120, and 300 s. The microscope images indicate that the penetration and movement of the particles are similar to each other, and the numerical predictions of rigid particles, using the constant zeta potential value for the particles, agree with the experimental observations. To show this behavior quantitatively, we determined the penetration depth of the particles (Figure 6). The experimental results are in close agreement with the numerical simulations. This is due to the zeta potential of the 1 BL and 4 BLs coated particles being almost identical (Figure 5B). Thus, we can conclude that the expressions for the rigid particles can explain the diffusiophoretic behavior of polyelectrolyte-coated particles of at least up to 4 BLs for our system.

### 4.4 Polyelectrolyte Coating of Various Particles

To study the effect of the initial particle surface on the polyelectrolyte adsorption and the resulting diffusiophoresis, we used two different particles: PS particles with sulfate-terminated groups and PS particles with Rhd-PEG (rhodamine-terminated group coated with polyethylene glycol). The zeta potential of PS particles with sulfate-terminated groups is almost constant (∼−68 mV) in the range of 1–10 mM NaCl. However, there is a large spread in values (Figure S7A). Feick et al.^21^ previously showed that sulfonated polystyrene latex has a nonuniform surface charge and that this effect could be reduced by 80% by polyelectrolyte or ionic surfactant adsorption. The zeta potential of PS particles with Rhd-PEG groups is strongly influenced by the electrolyte concentration^15^ (also Figure S7B). Here, we show the results of the polyelectrolyte adsorption (1 BL of PDADMAC/PSS coating with 25 mM NaCl) and the diffusiophoretic behavior of these particles with the numerical predictions.

We characterized the zeta potential of particles before and after coating with 1 BL of PDADMAC/PSS. Figure S7 shows the influence of the polyelectrolyte coating on the zeta potential of the various particles at different NaCl concentrations. The spread in the measured zeta potential of PS with sulfate-terminated particles can be significantly reduced by adsorbing only 1 bilayer of polyelectrolyte (according to the *F*-test at 95% confidence, *F* ≫ *F*_crit._). Similarly, the zeta potential value dependence with salt concentration for PS particles with Rhds-PEG groups can be reduced by adsorbing polyelectrolytes. Thus, polyelectrolyte adsorption can reduce the concentration dependence as well as the inherent variation in the zeta potential of particles.

It is important to emphasize that the final zeta potential value depends on the particle type, even though the final layer contains the same dissociating group (sulfate) in all cases. This is due to the different surface charges in the initial stage, which influence the total adsorbed charge. Pfau et al.^68^ showed that using polystyrene and mica substrates resulted in different heights and structures of single-layer PEI adsorption based on AFM measurements due to different initial surface charge densities. The adsorbed amount also changes depending on the surface charge density,^25,69^ which further leads to different zeta potential values. Moreover, the layer properties are dominated by the substrate (in our case the particle charge) since only 1 BL is adsorbed on the particle surface.^34^

**Figure 7:**
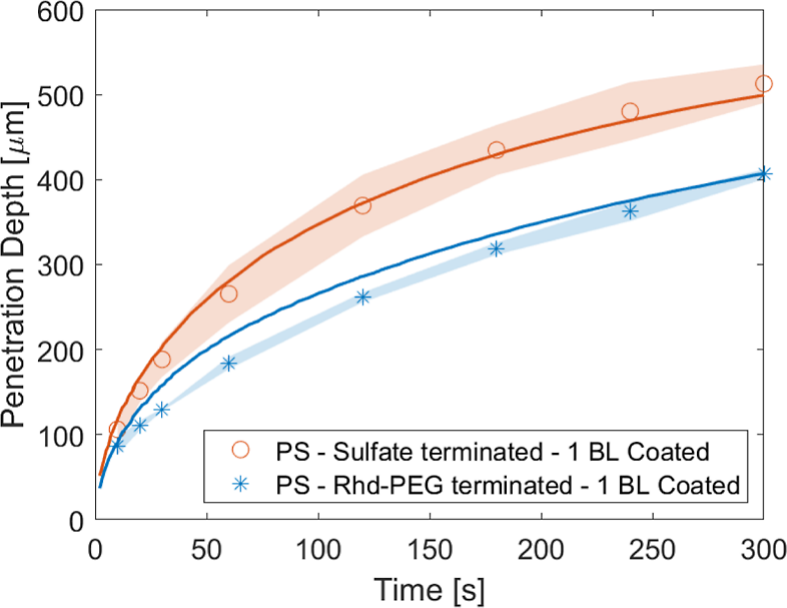
Diffusiophoretic behavior of PS particles with sulfate and Rhd-PEG-terminated groups that were subsequently coated with 1 BL. Results of the diffusiophoresis experiments (as penetration depths), with their corresponding simulations. The solid line represents the prediction of simulation where particle concentration is >0.1. The shadow area represents the 95% confidence interval of at least three measurements.

We analyzed the diffusiophoretic behavior of these two types of particles after coating them with 1 BL of PDADMAC/PSS in 25 mM NaCl. We found that the zeta potential value determines the penetration depth (Figure 7), as observed above. The adsorbed layer affects the diffusiophoretic behavior only through changes in the value of the zeta potential. Moreover, the zeta potential value is constant in the experimental salt concentration range; therefore, the constant potential assumption is valid.

## 5 Conclusions

Polyelectrolytes (PDADMAC/PSS) were adsorbed on various particle surfaces to observe the resulting changes in the zeta potential value and the corresponding diffusiophoretic behavior. We found that the final zeta potential of the particles was influenced by the salt concentration used during the PDADMAC/PSS coating process. The amount of adsorbed PSS and the available excess charged groups on the surface are influenced by the salt concentration during the coating process, resulting in different zeta potential values. After coating with 1 bilayer, the particles showed constant zeta potential values in the range of 1–10 mM NaCl due to the structural change of the polyelectrolyte with bulk salt concentrations and the strongly charged surface group of PSS sulfonate. We tested the diffusiophoretic behavior of the coated particles in a dead-end channel. The penetration depth of the particle through the dead-end channel increases with the absolute zeta potential value as a consequence of the higher diffusiophoretic mobility. The experimental observations were compared to simulations, which showed that the constant zeta potential assumption holds for the coated particles. Additionally, experiments were repeated for the multilayer system and various particles. The additional coating did not affect the zeta potential value and, therefore, the diffusiophoretic behavior. The initial surface charge influences the polyelectrolyte adsorption and the resulting zeta potential value. The constant zeta potential assumption applies to all systems, which was not the case when there was no coating present. The study also showed that polyelectrolyte coatings can reduce salt concentration dependence of zeta potential, tune particle zeta potential, and reduce the variation in particle zeta potentials.

## Supporting Information Available

Supporting Information includes information about the PSS calibration to determine the adsorption amount, zeta potential value of the uncoated and coated particles, the effect of particle concentration value in the simulation on the results, and effective particle diffusivity fitting details (PDF)
