# Supplementary material for: Diffusiophoretic Behavior of Polyelectrolyte-Coated Particles
Source: Langmuir. 2024 Mar 7;40(11):5934–44. doi: 10.1021/acs.langmuir.3c03916 (PMC10956496; doi:10.1021/acs.langmuir.3c03916)
Supplement: Supplementary file 1 — la3c03916_si_001.pdf [file la3c03916_si_001.pdf]

Supporting Information: Diffusiophoretic Behavior of Polyelectrolyte Coated Particles

Burak Akdeniz, Jeffery A. Wood,* and Rob G. H. Lammertink*

Soft Matter, Fluidics and Interfaces, University of Twente, MESA+ Institute for Nanotechnology, P.O. Box 217, 7500 AE Enschede, The Netherlands

E-mail: j.a.wood@utwente.nl; r.g.h.lammertink@utwente.nl

Phone: +31 (0)534892961; +31 (0)534892063

Number of pages: 9

Number of figures: 7

Number of tables: 1

Contents

S1 Experimental Setup **2**

S2 PSS Concentration - Calibration Curves **3**

S3 Particle Zeta Potential with Varied Electrolyte Concentration **4**

S4 Particle Concentration/Number Value **5**

S5 Effective Particle Diffusivity - Fitting **7**

S6 Zeta Potential Values of PS - Sulfate Terminated and PS - Rhd- PEG Terminated Particles with Varied Electrolyte Concentration **9**

S1 Experimental Setup

The experimental setup, the dead-end channel setup, is shown in Figure S1. The picture of the whole system is shown in Figure S1 A, which also shows the connected tubing (particle suspension flows here). The tubing is connected to the main channel shown in the grey area in Figure S1 B schematically. In the experiments, we focused on the dead-end channel, which is blue in colour. The dimensions of the system are also given in the figure. A 2-D representation of the system is given in Figure 1.

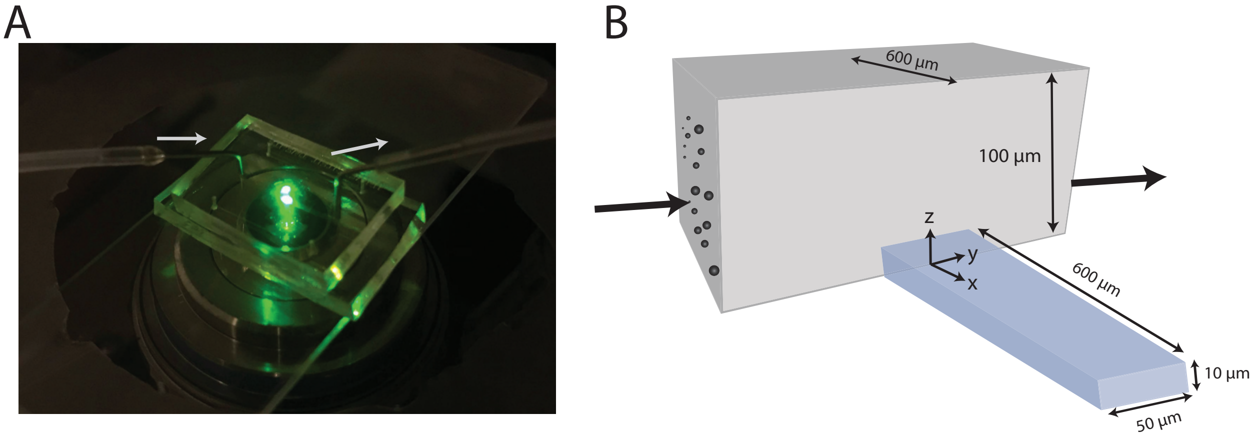

Figure S1: The experimental system. (A) The picture of the whole experimental setup. (B) 3-D schematic of the dead-end channel and its dimensions. For the 2D image, please refer to Figure 1.

S2 PSS Concentration - Calibration Curves

Calibration curves were obtained with known concentrations of PSS for varied salt concentrations. The calibration curve was repeated for every electrolyte concentration since the properties of polyelectrolytes change with salt concentrations. The PSS amount in the supernatant is determined by using the calibration curve. In the calculation, we ignored the adsorption on the centrifugal tube wall due to the low surface area compared to all particles. However, the determined values were in error margins of determination.

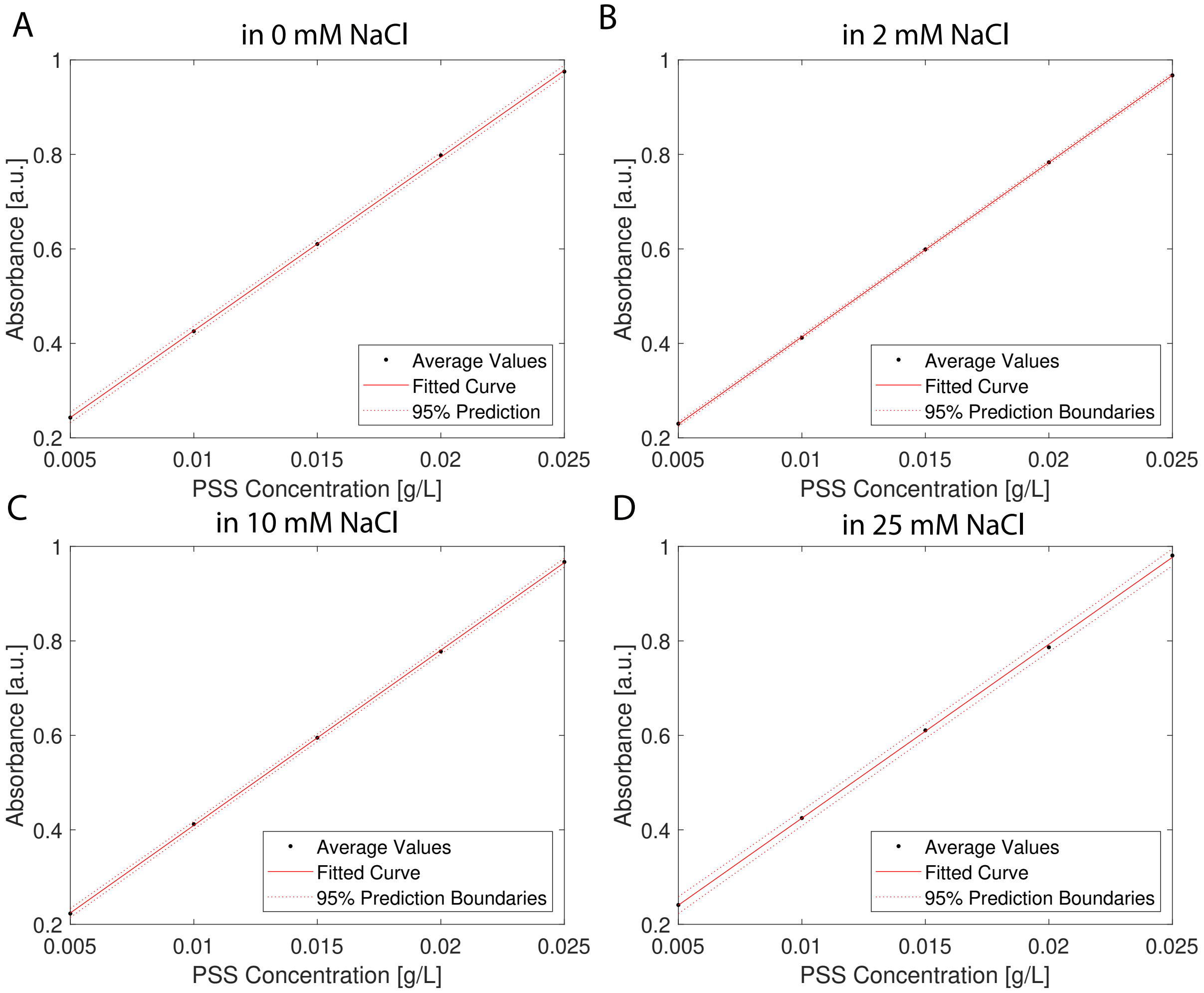

Figure S2: The calibration curves for the PSS absorbance at 225 nm. The background salt concentration is (A) 0 mM, (B) 2 mM, (C) 5 mM, and (D) 10 mM NaCl.

S3 Particle Zeta Potential with Varied Electrolyte Concentration

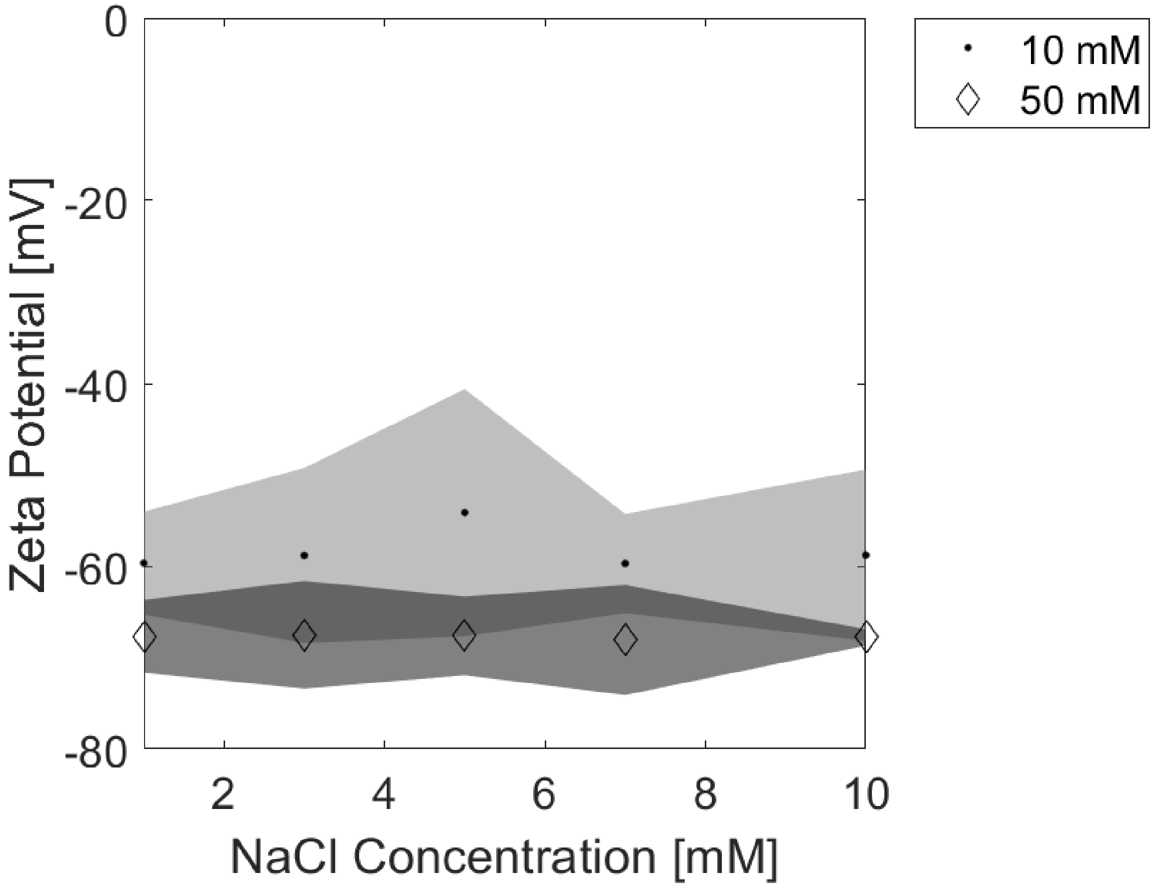

Figure S3: The zeta potential of the coated particles with respect to NaCl concentration in solution during zeta potential measurement. The legend indicates the electrolyte concentrations that were used in the coating process. Other electrolyte concentrations are provided in Figure 2 in the main text. The shadow areas represent the 95% confidence interval of three separate coating experiments.

S4 Particle Concentration/Number Value

The result of the advection-diffusion equation gives a concentration value for particle concentration at each stage and position. Throughout the paper, we have used the particle 'concentration' values > 0.1. In Figure 4, we measured the particle concentration value at the center of the dead-end channel $x = 25$ µm, $z = 5$ µm, and $y = 0$-600 µm. Then, we determined the position value where the particle concentration values start to be > 0.1 in each time interval. Especially for the low zeta potential values, we observed that the particle concentration > 0.1 assumption overestimates the experimental observations for the early stages. Using the same approach, we determined the positional results with > 0.5 values and plotted each result in Figure S4 with experimental observations. The experimental observations and theoretical predictions show similar behavior, especially in the early stage when the particle concentration value is > 0.5. The result of the advection-diffusion equation gives a probability of the particle. This approach does not take into account the particle size, the particle-particle interaction and the particle-wall interaction. However, this approach gives the possible locations of the particle.

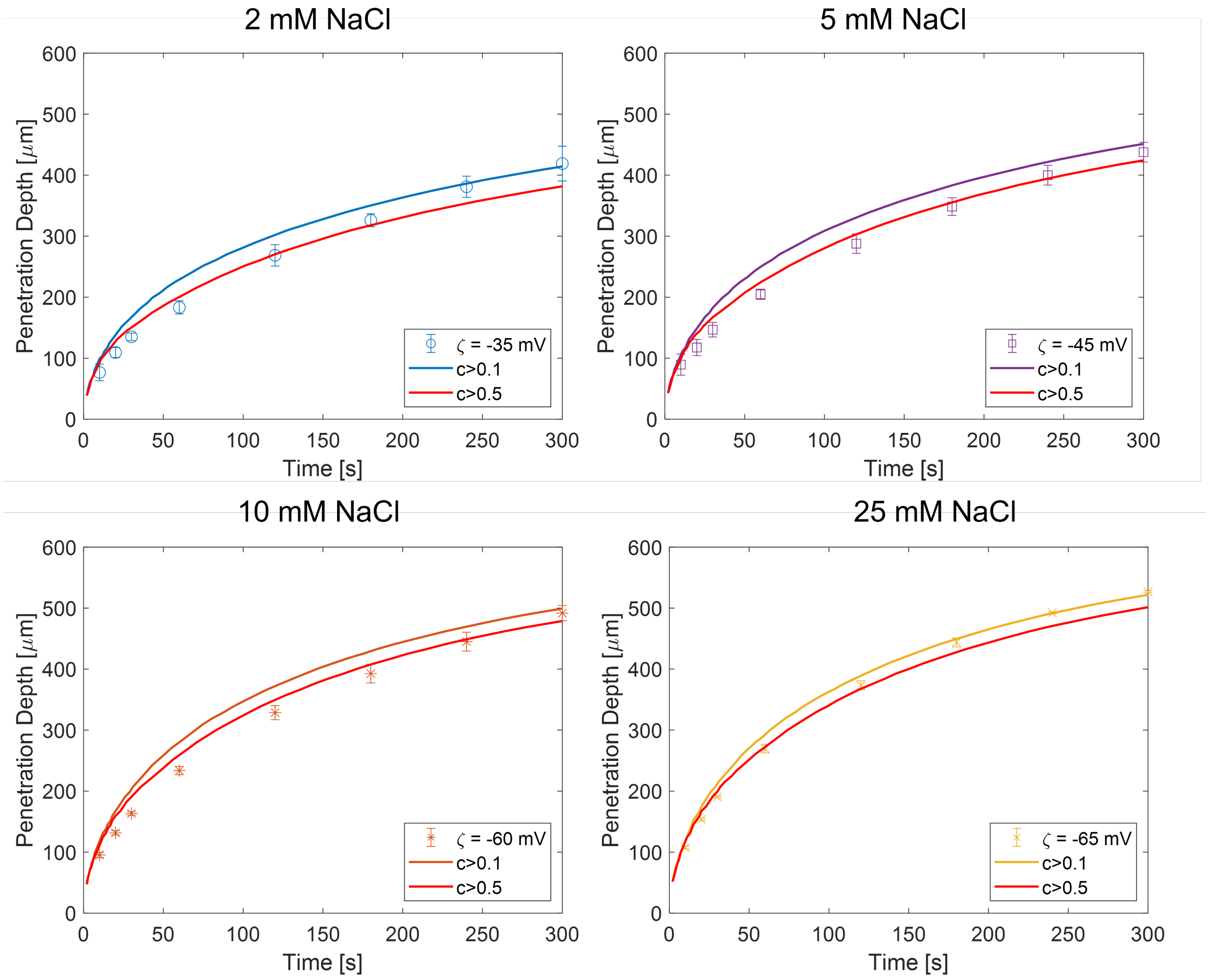

Figure S4: The penetration depth of the PS-carboxylate particles coated with 1 BL of PDADMAC/PSS. The salt concentrations during coating are given at the top of each figure; 2 mM NaCl, 5 mM NaCl, 10 mM NaCl, and 25 mM NaCl. Every figure contains the experiment observation (shown in markers), and solid lines show the particle concentration $c_p > 0.1$ (same color as the marker) and $c_p > 0.5$ (red color).

S5 Effective Particle Diffusivity - Fitting

To measure the effective particle diffusivity (D_{eff}) in a dead-end channel, we fit an equation $\Delta x = \sqrt{2 \cdot D_{\text{eff}} \cdot t}$. For the fitting, we have used Curve Fitting Tool in MATLAB 2021 (Mathworks, California, United States). For experimental (Figure S5) and theoretical fit (Figure S6) with 95% confidence bounds. The values of the fitted parameters are given in Table 1 and Table S1. ± values in the table show the 95% confidence level. In Table S1, there are two effective diffusion coefficients (D_{eff1} & D_{eff2}), which were calculated penetration depth for particle concentration c > 0.5 and c > 0.1, respectively.

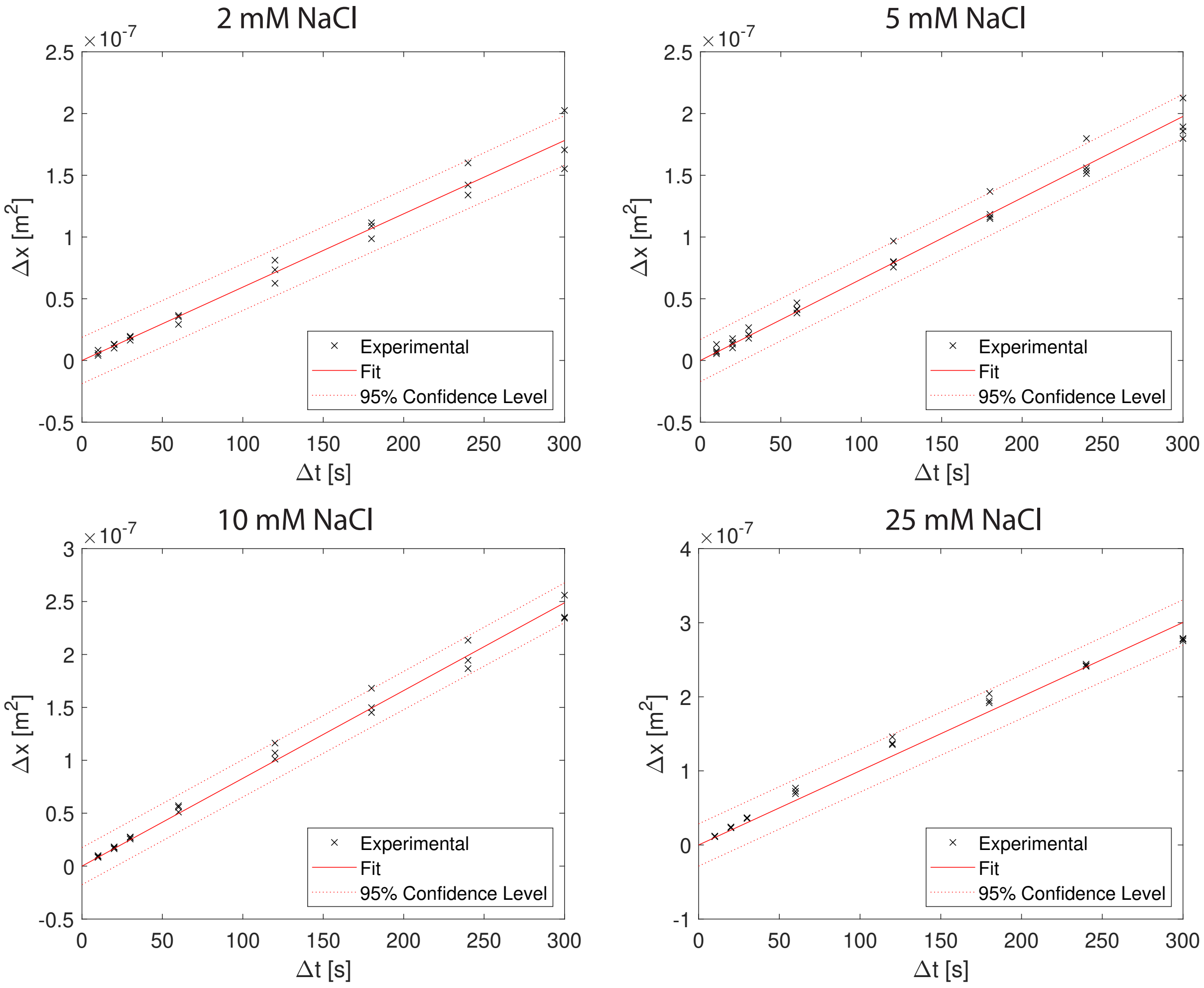

Figure S5: Effective particle diffusivity value of the averaged experimental values. The salt concentrations during coating are given at the top of each figure.

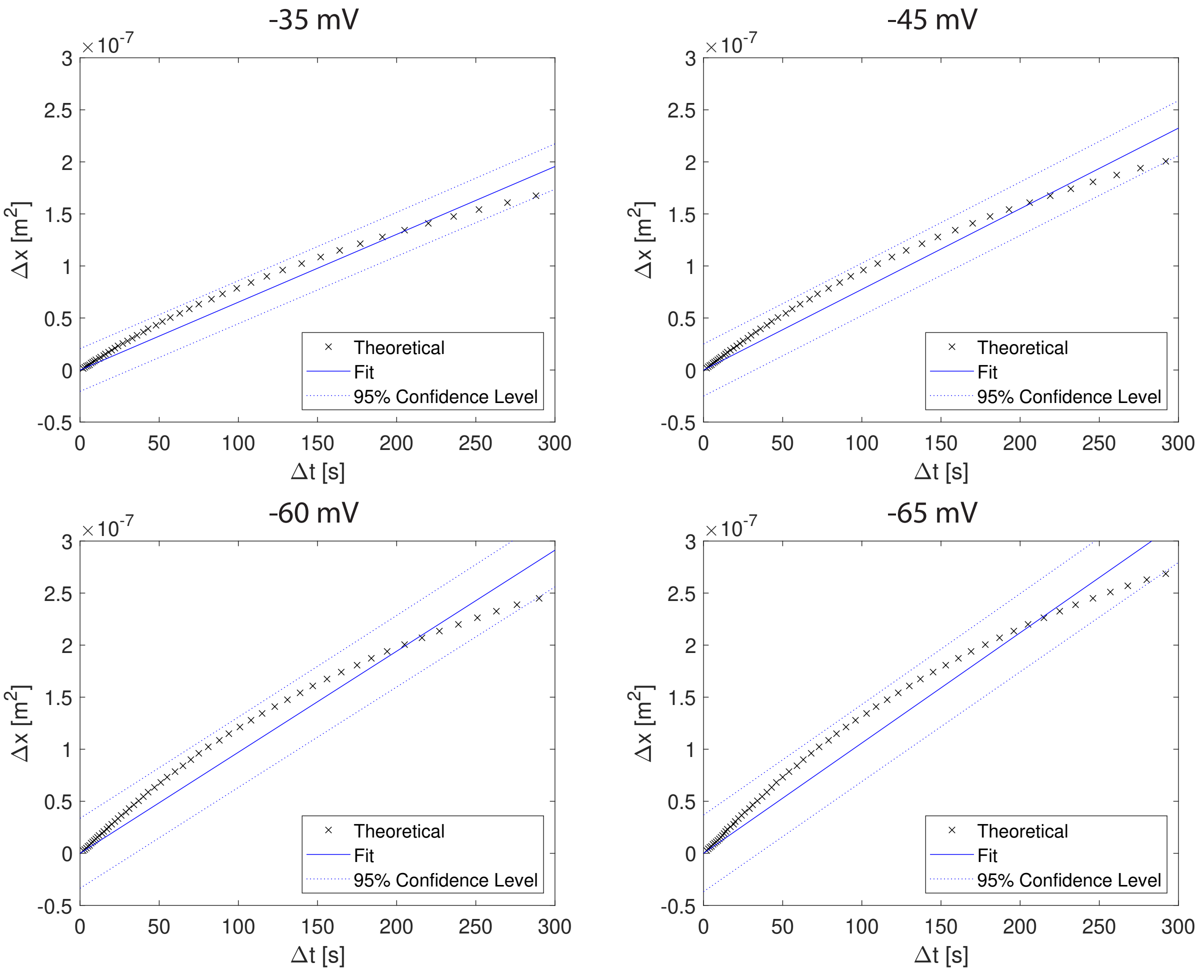

Figure S6: Effective particle diffusivity value of the predicted theoretical values. The zeta potential values of the particles are given at the top of each figure.

Table S1: Effective diffusion coefficient of the 1 BL polyelectrolyte coated particles. The salt concentration during coating (c_{salt}) is given with the representative averaged zeta potential values ($\zeta_{\text{avg.}}$). The values after $\pm$ show the 95% confidence level of the fit.

c_{salt} [mM]	$\zeta_{\text{avg.}}$ [mV]	$D_{\text{eff1}} \cdot 10^{10}$ [m^2/s]	$D_{\text{eff2}} \cdot 10^{10}$ [m^2/s]
2	-33.8 $\pm$ 1.4	2.70 $\pm$ 0.08	3.26 $\pm$ 0.12
5	-47.6 $\pm$ 3.3	3.37 $\pm$ 0.10	3.87 $\pm$ 0.14
10	-57.1 $\pm$ 4.7	4.37 $\pm$ 0.14	4.85 $\pm$ 0.18
25	-64.9 $\pm$ 1.5	4.85 $\pm$ 0.15	5.30 $\pm$ 0.18

S6 Zeta Potential Values of PS - Sulfate Terminated and PS - Rhd- PEG Terminated Particles with Varied Electrolyte Concentration

Zeta potential values of the bare and 1 BL PDADMAC/PSS (in 25 mM NaCl) coated particles are shown in Figure S7. After coating, the particles were placed in different NaCl solutions for the zeta potential measurement.

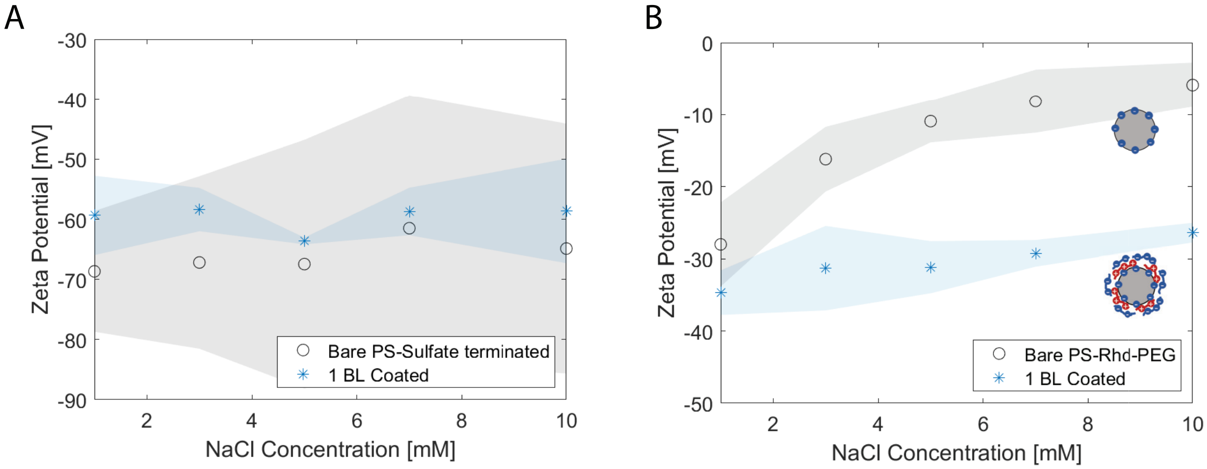

Figure S7: Characterization of PS particles with sulfate terminated, and PS with Rhd-PEG groups. The zeta potential of (A) PS particles with sulfate terminated and (B) PS with Rhd-PEG groups are given before and after coating with 1 BL of PDADMAC/PSS in 25 mM NaCl. The shadow area represents the %95 confidence interval of three separate coating experiments.
